# A Smart pH-Responsive Three Components Luminescent Hydrogel

**DOI:** 10.3390/jfb7030025

**Published:** 2016-09-12

**Authors:** Yibao Li, Wei Liu, Linxiu Cheng, Ping Huang, Yu Peng, Yongquan Wu, Xun Li, Xiaokang Li, Xiaolin Fan

**Affiliations:** Key Laboratory of Organo-pharmaceutical Chemistry, Gannan Normal University, Ganzhou 341000, China; liuw@gnnu.cn (W.L.); chenglx@gnnu.cn (L.C.); huangp@gnnu.cn (P.H.); pengy@gnnu.cn (Y.P.); wuyq@gnnu.cn (Y.W.); lix@gnnu.cn (X.L.); fanxl2013@gnnu.cn (X.F.)

**Keywords:** hydrogel, pH-response, luminescence

## Abstract

In this study, we report a novel three-component luminescent hydrogel, which is composed of amino acid derivatives (*N*,*N*′-di valine-3,4,9,10-perylenetetracarboxylic acid, NVPD), riboflavin (RF), and melamine (MM). The three-component hydrogel is attributed to multiple hydrogen bonds and the strong π-π stacking interaction between these molecules. Based on the strong hydrogen bonding of the gelator, when the reversible process between the gel and the solution take places it changes the pH of the system from 6.1 to 10.6. In addition, green fluorescence could be the emissive of the hydrogel under 498 nm and the conversion process of the aggregation state repeated reversibly by altering the value of ambient pH. This pH-responsive luminescent gel may display potential for use in nano pH sensors.

## 1. Introduction

Supramolecular hydrogels driven by non-covalent interactions of inter-molecules such as hydrogen bonding [1,2,3], host-guest recognition [4], electrostatic interaction [5], aromatic stacking [6,7], or metal-ligand coordination [8,9,10] have great applications in mechano-responsive sensor materials [11], drug delivery vehicles [12,13,14,15], biomedicine [16,17], cell culture [18,19], vitreous substitutes [20] and shape memory materials [21]. The dynamic nature of the non-covalent interactions can be exploited to design adaptive systems, because the environmental sensitivity of non-covalent bonds makes the systems willingly respond to external stimuli [22]. Compared with covalent polymers, the supramolecular hydrogels are more sensitive to watch for their thermo-reversibility and stimuli-responsiveness. In addition, the multiple non-covalent bond interaction between the gelater can build a robust system [23,24]. Recently, a large number of stimuli-responsive hydrogels has been reported [25,26,27,28]. One application prospect approach is the use of small-molecule self-assembly for the preparation of “smart” materials [29]. It is still a challenge which utilized small molecules to form a robust system to respond to an external stimulus.

As one of the non-covalent bonding effects, the hydrogen bonding interaction plays an important role in the formation of the gel. Compared with copolymer counterparts, the molecules which can assemble via non-covalent interactions are captivating for fabrication and reversibility [30]. The non-covalent interactions are advantageous for the creation of various kinds of smart materials due to their dynamic character [31]. Over the last decades, the smart or responsive materials met the demand of diverse and dynamic material properties that are able to change form or function in response to cues such as pH, temperature, electric field, enzymes, etc. [32,33,34,35,36,37]. Among the stimuli-responsive hydrogels, the pH-sensitive hydrogel is well studied, where hydrophilic networks undergo volume deformations in response to changes in the surrounding pH [38]. The pH-responsive hydrogel which responds to the change in pH values in the environment is due to the gelator being able to dissociate or associate with hydrogen ions as the pH changes in the environment. The process changing from solution to gel leads the assignment of molecules from a disorderly state to orderly, in which the self-assembled hydrogel may undergo a reversible sol-to-gel or gel-to-sol transition [39,40].

In this paper, we reported a smart pH-responsive three-component luminescent hydrogel which is made up of amino acid derivatives, riboflavin, and melamine. Perylene bisimide, one of the organic dyes, has been proven for an application prospect in organic light-harvesting systems, n-type channel field effect transistors, fluorescent emitters and biosensors, and in the construction of self-assembling nanostructures as an active components [41,42,43,44,45,46,47,48,49,50,51,52,53,54,55,56]. Such as, Rybtchinski and co-workers have reported a stimuli-responsive supramolecular gel of perylene derivatives [57]. A new type of amphiphilic molecule was synthesized, forming the amido between the valine and 3,4,9,10-perylenetetracarboxylicdiimide molecules. We obtained a new type of fluorescent gel formed under ultrasound at room temperature between NVPD, RF, and MM via hydrogen-bond and π-π stacking; this finding has not ever been reported. The gel is able to respond to the pH change from the system. Moreover, it can be reversible with the system’s pH change. 

## 2. Materials and Methods

### 2.1. Materials

All starting materials were purchased from commercial suppliers and used without any further processing.

### 2.2. Synthesis of NVPD

0.392 g (1.0 mmol) perylene-3,4,9,10-tetracarboxylic dianhydride, 0.330 g (2.0 mmol) l-valine and 2.0 g imidazole were heated at 90 °C for 40 min under nitrogen atmosphere. When the imidazole was melting, the temperature was raised to 120 °C for 6 h. Then 25 mL of CH_3_CH_2_OH was poured into the round-bottom flask, refluxed for 6 h at 90 °C, cooled down at room temperature, then dropped 1.0 moL/L HCl into the solution until the mixture solution was acidity. Keeping the solution in ambient temperatures overnight to let it precipitate out. The precipitate was filtered and washed with H_2_O. The product was dried at room temperature to get mulberry powder (yield: 0.57 g, 87%). The structure and purity of the product were confirmed by ^1^H-NMR, FT-IR and MS.^1^H-NMR (DMSO, 400 MHz, ppm): 8.61–8.62 (d, 4H), 8.42–8.44 (d, 4H), 5.18–5.20 (d, 2H), 2.70–2.76 (m, 2H), 1.26–1.27 (d, 6H), 0.78–0.79 (d, 6H); IR (KBr): 1252.5, 1401.7, 1591.9 cm^−1^ (C=C), 1342.4 cm^−1^ (–CH_3_), 1656.6, 1698.3, 1737.9 cm^−1^ (C=O), 2963.9 cm^−1^ (C–H), 3460.3 cm^−1^ (O–H); MALDI-TOF-MS: calcd for C_34_H_26_N_2_O_8_, 590.0.

### 2.3. Preparation and Characterization of Gelation

The gelation test of the amino acid functionalized perylene derivatives (NVPD) is carried out using a test tube inversion method at room temperature. To prepare the gels system, a weighed sample of amino acid functionalized perylene derivatives at certain molar ratio (NVPD/RF/MM = 1/2/2) is put into a septum-capped vial (5 mL) with 400–500 μL various organic solvents. The mixture is heated to 50–60 °C, cooled to room temperature and left for a period of time. If no flow is observed when inverting the vial, it is considered to be a gel. The critical gelation concentration (CGC) of the gel was 0.57% W/V, determined by measuring the minimum amount of gelators for the formation of a stable gel at room temperature. The driving forces for gelation will be discussed below.

NMR spectra were measured on a Bruker ULTRASHIELD 400 (Bruker, Zürich, Switzerland) spectrometer. The luminescence spectra were measured on a LS55 fluorescence spectrophotometer (PerkinElmer, Billerica, MA, USA). The path length of the quartz cell was 1 cm, while the emission bandwidth was 5 nm. The FT-IR spectra were recorded on an AVATAR 360 FT-IR spectrophotometer (Nicolet, Madison, WI, USA). The powdered samples were mixed with KBr to prepare the thin films in the solid-state FT-IR studies. SEM images of these samples were recorded using FEI QUANTA 450 (FEI, Hillsboro, OR, USA) with accelerating voltage 25.0 kV. Samples were obtained through dropping the gels on a flat surface of a cylindrical aluminum substrate and allowed to dry at room temperature. Then the samples were coated with gold using a MSP-1S Magnetron Sputter Coater (Tokyo, Japan). Rheological characterization was performed using a stress-controlled rheometer (HAAKE RheoStress 6000, Offenburg, Germany) with parallel plate type geometry (plate diameter, 3.5 cm). A solvent trap equipped with rheometer was used to protect the sample from evaporation. Frequency sweeps at selected temperatures were carried out over a range of 0.1–100 rad·s^−1^ at strain amplitude of 1%.

## 3. Results and Discussion

**Figure 1:**
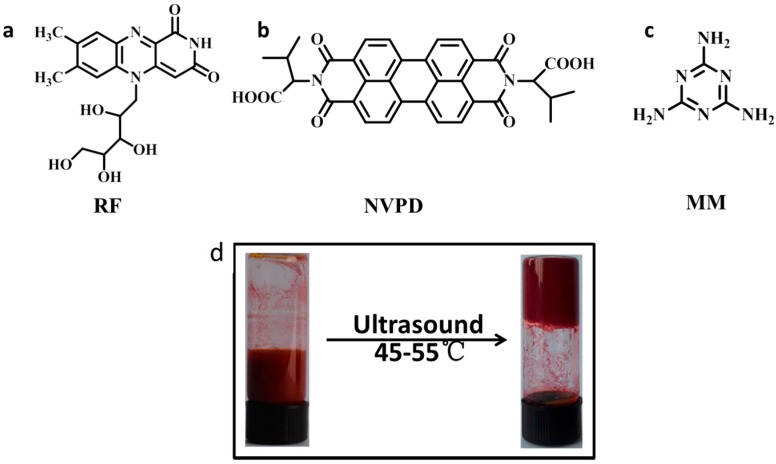
The chemical structure of gelators (**a**) RF; (**b**) NVPD; (**c**) MM; (**d**) optical image of the prepared hydrogel.

The structures of the NVPD, RF, and MM are shown in Figure 1, respectively. As shown in Figure 1, the NVPD contains two valine residues and a perylene core. The residues may be contributed to the gelation process of the hydrogen bonds. In addition, the perylene core is an interesting class of chromophores and fluorophores on account of its enhanced stability, good optical properties and pronounced capabilities of self-assembly by means of π-π stacking, which may play an important role in functional devices. According to the method of gelation described in the Experimental Section, we tested different ratios of components. It was found that the molar ratio of 1:0.3:2, 1:1:2, 1:2:2, and 1:2:3 of NVPD/RF/MM can form a hydrogel under ultrasound at room temperature in a mixture solution (THF/H_2_O = 5/5, Figure S1). NVPD has a perfect solubility in tetrahydrofuran (THF), but it is insoluble in water. Any two components of the three components cannot form a hydrogel in a similar condition (Figure S2). Besides, the three-component hydrogels were unable to form under similar conditions expect ultrasound (Figure S3). The three components were transferred to the mixture solution ultrasonically after 50 min (pH 6.1), and the hydrogel was formed (Figure 1d). The flowability of mixtures (the mole ratio of NVPD:RF:MM = 1:2:2) is greatly reduced and the gels were formed after ultrasound at 45–55 °C.

**Figure 2:**
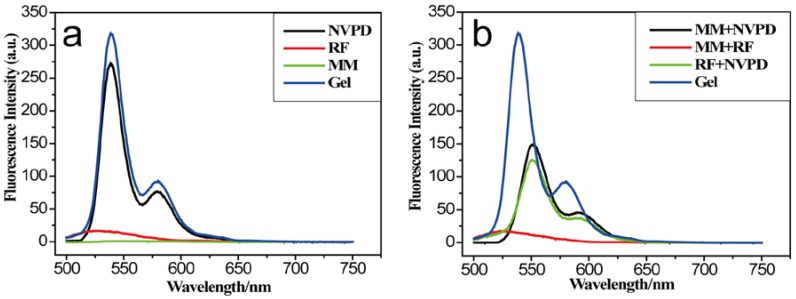
Fluorescence spectra of solution and gel at room temperature (λex = 490 nm, path length = 5 mm). (**a**) Three components of gel and one-component solution ([NVPD] = 10^−6^ mol/L, [RF] = 2 × 10^−6^ mol/L, [MM] = 2 × 10^−6^ mol/L); (**b**) Three components of gel and two-component solution ([NVPD] = 10^−6^ mol/L, [RF] = 2 × 10^−6^ mol/L, [MM] = 2 × 10^−6^ mol/L).

The fluorescence of the three-component hydrogel and the single components was investigated (Figure 2). Perylene bisimides have near-unity fluorescence (FL) quantum yields in diluted solutions and fluorescence quenching in the state of aggregation, due to dipole-dipole interactions and/or π-π stacking between molecules [58]. In fluorescence spectra, the fluorescence intensity of gels was higher than NVPD in the dilute solution ([NVPD] = 10^−6^ mol/L, [RF] = 2 × 10^−6^ mol/L, [MM] = 2 × 10^−6^ mol/L). The gelator could form micelles by hydrogen bonding in the mixture solution. In addition, the fluorescence would be quenching due to the π-π stacking. However, the three-component gelator formed new aggregates, and the steric effects were stronger than NVPD. This made the intensity of the gel stronger than that of the NVPD. Compared with the gel, any two components of the hydrogel had lower fluorescence intensity, as shown Figure 2b. Moreover, the maximum absorption peak of any two components appeared to red-shift. Two components between molecules get together to form polymers, the size of which was smaller and the inter-molecular π-π stacking of which was stronger. It made the fluorescence quenching. In addition, the fluorescence of the hydrogel is investigated under a 365 nm ultraviolet lamp (Figure S4). It is interesting that the fluorescence appeared with the hydrogel and disappeared with the solution at 365 nm. The result is consistent with the fluorescence spectrum. The steric space between the new aggregate was bigger, the π-π stacking effect was weakened, and then fluorescence appeared at 365 nm. The infrared spectroscopy provides some important evidence of interaction between the molecules. The strong C=O bands at 1750 cm^−^^1^ and 1698 cm^−^^1^ in pure NVPD were observed, which indicated that there was no strong hydrogen bond between molecules. It means that there was no strong hydrogen bonding interaction between inter-molecules. The single bond at 1695 cm^−^^1^ was observed in xerogel (Figure S5). It was ascribed to the hydrogen-bonded C=O vibration. This phenomenon shows that the hydrogen bonds are fit to form the gel [59,60]. The result shows that the intermolecular interaction plays an important role in the formation of the gel. 

**Figure 3:**
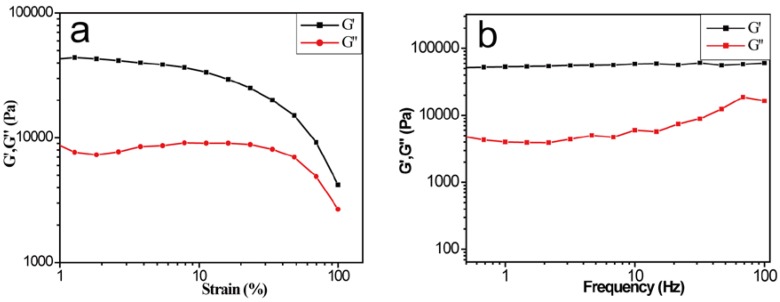
(**a**) Strain sweep of the three-component gel at the frequency of 5.6 rad·s^−^^1^; (**b**) Frequency sweep of the gel at a strain of 0.1%.

In order to explore the mechanical properties of the gel, rheological tests were carried out by the rheometer. The test results are shown in Figure 3a,b; the storage modulus (G’) was much higher than the loss modulus (G”) between 1%–100% under a certain strain. In the linear viscoelastic region, the value of G’ for the NVPD/RF/MM gel was over 3 × 10^4^ Pa, showing that the gel has excellent mechanical properties (Figure 3a). In addition, a frequency sweep was performed (Figure 3b), and values of the three components indicate that the storage modulus (G’) dominated the loss modulus (G”). It means that the hydrogel has wide potential applications in mechanical and medical fields.

**Figure 4:**
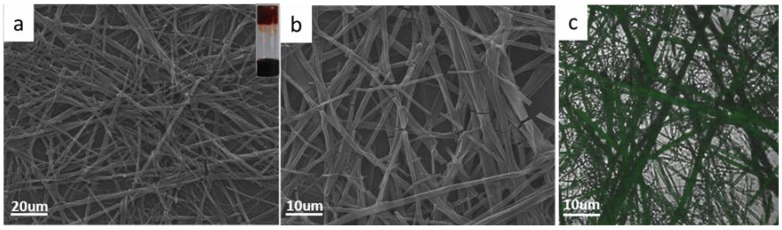
(**a**) The xerogels in mixed solution for larger scale [insert image was photo of the gel]; (**b**) NVPD/RF/MM hydrogel in mixed solution at small scale; (**c**) CLSM images of GP/RF/MM hydrogel (overlay image). ([NVPD] = 10^−3^ mol/L, [RF] = 2 × 10^−3^ mol/L, [MM] = 2 × 10^−3^ mol/L).

Furthermore, the surface morphology of the dry hydrogel was observed by scanning electron microscopy (SEM). Nanofibers were observed, as shown in Figure 4a,b. The long and slender fibers interweave together, leading to the formation of a 3D mesh structure. It means that the gel has a potential application in drug sustained release. The optical image of the hydrogel was shown in Figure 3a (inset). Additionally, the fluorescence of the hydrogel could be observed under a confocal laser scanning microscope (CLSM) upon excitation at 409 nm. The long and thin fibers tangled together with high fluorescent intensity as shown Figure 4c. It means that the hydrogel has strong fluorescence.

**Figure 5:**
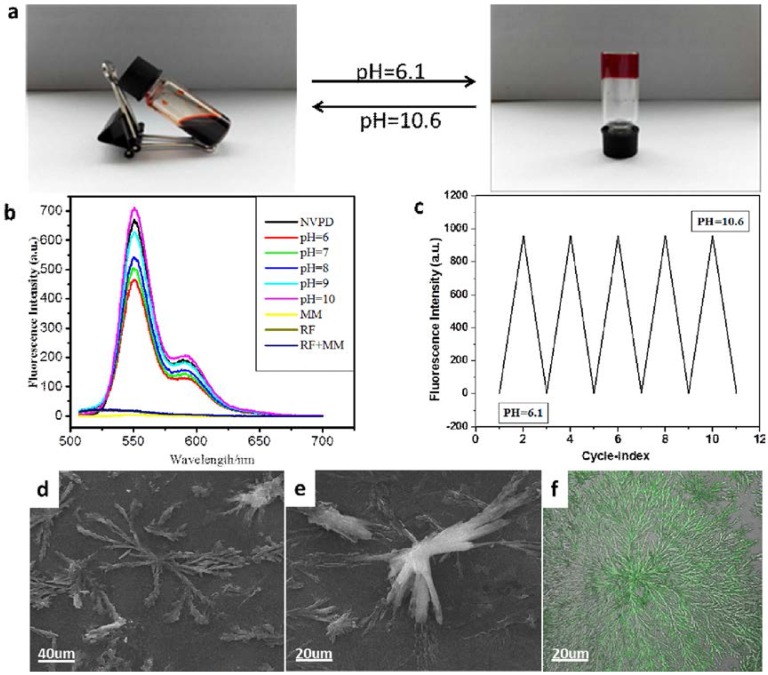
(**a**) Optical images of the gel’s reversible process by adjusting the pH; (**b**) Fluorescence spectra of solution at room temperature of the NVPD system at different pHs are shown. Typical molar ratio = 1:2:2, the NVPD concentration is 10^−6^ M, at λex = 496 nm [path length = 5 mm]; (**c**) Turn cyclical change in fluorescence intensity at 496 nm of the dilute solution ([NVPD] = 10^−6^ M); (**d**) SEM images of the NVPD/RF/MM gel after treatment with alkali for larger scale ([NVPD] = 10^−3^ mol/L, [RF] = 2 × 10^−3^ mol/L, [MM] = 2 × 10^−3^ mol/L); (**e**) SEM images of NVPD/RF/MM hydrogel in mixed solution at small scale after treatment with alkali; (**f**) CLSM images of GP/RF/MM hydrogel.

We further tested the reversibility of the gel system and found that the gel has excellent reversibility by pH adjustment. The NVPD/RF/MM (the mole ratio was 1:2:2) hydrogel could be constructed at pH 6.1. Then, the gel was dissolved when the alkali liquor (50 μL) joined in and the gel was back with fluorescent regeneration by adjusting the pH to 6.1, as shown in Figure 5a. However, the hydrogel was unable to form gel again after the addition of the alkali liquor in which the mole ratio was 1:2:2 (Figure S6). The sol-to-gel and gel-to-sol cycle could be repeated many times when the pH value was changed from 10.6 to 6.1 (Figure 5c). To detect the fluorescent gel performance, the fluorescence spectrum was used to investigate the gel fluorescence properties in different pH solutions, as shown in Figure 5c. Compared with these curves, the fluorescence intensity of NVPD/RF/MM (1:2:2) was increased with the pH increasing at 550 nm and 589 nm. The fluorescence intensity is the highest in the aqueous solution with a pH value of 10 (pink line) and the lowest at pH 6 (red line). This performance has potential application in detecting pH changes in the environment. In addition, we observed the gel microstructure with the pH value increasing to 12. The result showed that the microstructure changed. The nanofibers disappeared and rings were observed with the change of the pH value (Figure 5d,e). Moreover, the fluorescence still existed (Figure 5f).

**Figure 6:**
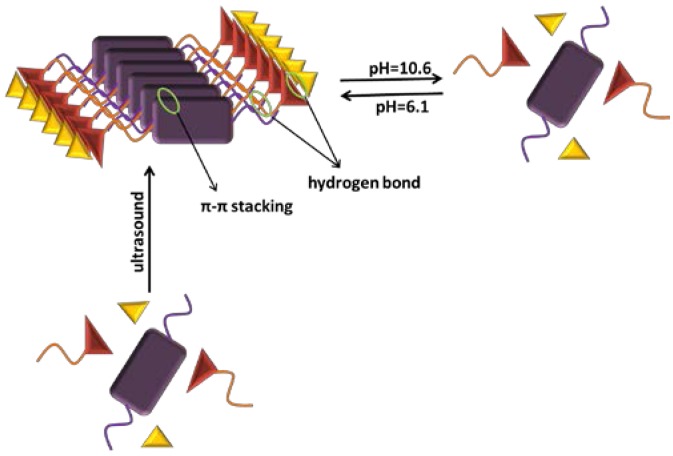
Proposed mechanism of self-assembly and pH-response process.

Based on the above experimental results, a model was proposed as shown in Figure 6. The RF and MM form three complementary NH–O and NH–N hydrogen bonds between diaminopyridine and diimide [61,62,63,64,65]. When the concentration of the gel factors reaches the minimum gelation concentration, the RF and MM molecules combined together via the hydrogen bonding and π-π interactions. If the system pH has been changed, the hydrogen bonding and the gel have been destroyed. 

## 4. Conclusions

We reported a three-component pH-responsive luminescent hydrogel made of amino acid derivatives, vitamin B_2_ and melamine in a mixture solution (THF/H_2_O = 5/5). The experimental results show that the three-component hydrogel was formed under ultrasound, derived by hydrogen bonding and π-π stacking. The smart hydrogel exhibited excellent mechanical properties and a pH-responsive luminescent reversible performance. We believe that the gel will provide potential applications in fluorescence and pH sensors.

## Supplementary Materials

The following are available online at http://www.mdpi.com/2079-4983/7/3/25/s1.
