# Supplementary material for: A Smart pH-Responsive Three Components Luminescent Hydrogel
Source: J Funct Biomater. 2016 Sep 12;7(3):25. doi: 10.3390/jfb7030025 (PMC5040998; doi:10.3390/jfb7030025)
Supplement: Supplementary file 1 [file jfb-07-00025-s001.pdf]

Supplementary Materials: A Smart pH-Responsive Three Components Luminescent Hydrogel

Yibao Li *, Wei Liu, Linxiu Cheng, Ping Huang, Yu Peng, Yongquan Wu, Xun Li, Xiaokang Li * and Xiaolin Fan

1. Photos of Different Mole Ratio Gels

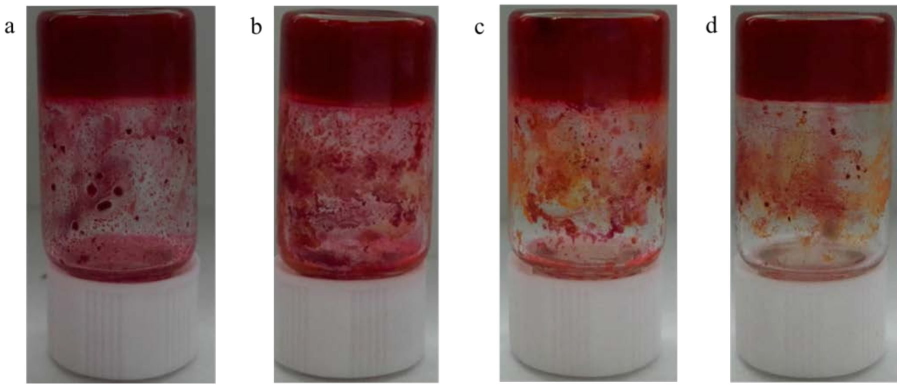

Figure S1. Optical images of the multicomponent gel for different mole ratio, (**a**) NVPD:RF:MM = 1:0.3:2, [NVPD] = 6.7 × 10^{-3} mol/L, [RF] = 2 × 10^{-6} mol/L, [MM] = 1.3 × 10^{-2} mol/L; (**b**) NVPD:RF:MM = 1:1:2, [NVPD] = 6.7 × 10^{-3} mol/L, [RF] = 6.7 × 10^{-3} mol/L, [MM] = 1.3 × 10^{-2} mol/L; (**c**) NVPD: RF : MM = 1:2:2, [NVPD] =6.7 × 10^{-3} mol/L, [RF] =1.3 × 10^{-2} mol/L, [MM] =1.3 × 10^{-2} mol/L; (**d**) NVPD:RF:MM = 1:2:3, [NVPD] = 6.7 × 10^{-3} mol/L, [RF] = 1.3 × 10^{-2}mol/L, [MM] = 2 × 10^{-2} mol/L.

2. Addition Photos of the Two-Component Gelator Solution

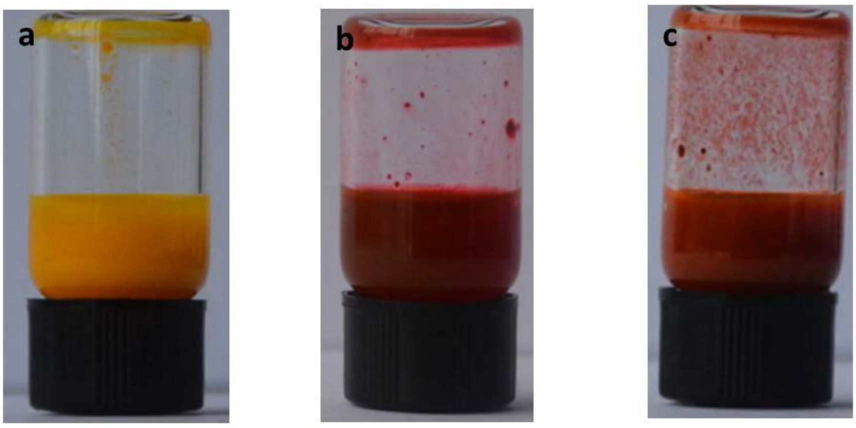

Figure S2. Optical images of the multicomponent gel for different ratio without ultrasound at the room temperature, (**a**) RF: MM = 2:2; (**b**) NVPD:MM =1:2; (**c**) NVPD: RF =1:2.

3. Addition Photos of the Three-Component Gel with Different Ratio

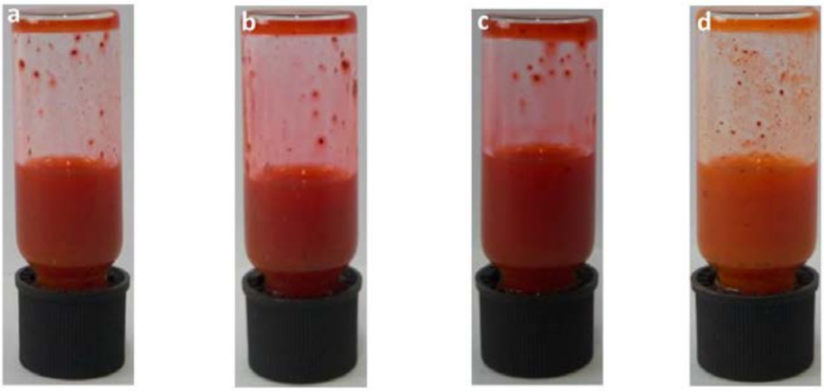

Figure S3. Optical images of the multicomponent gel for different ratio without ultrasound at the room temperature, (**a**) NVPD:RF:MM = 1:0.3:2; (**b**) NVPD:RF:MM = 1:1:2; (**c**) NVPD:RF:MM = 1:2:2; (**d**) NVPD:RF:MM = 1:2:3.

4. Addition Photos of the Three-Component Gel under 365nm UV Lamp with the Transition Gel-to-Sol Process

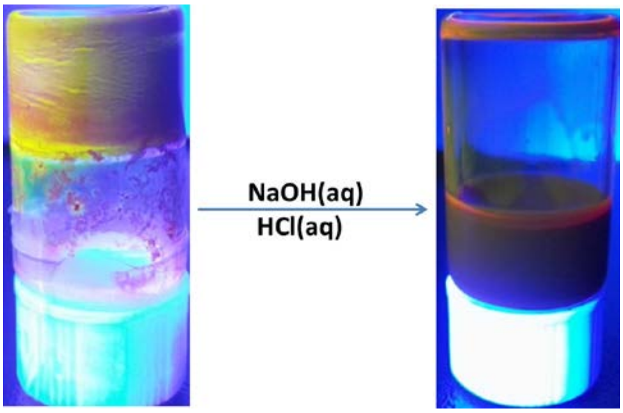

Figure S4. Fluorescence images of the gel ([NVPD] = 10^{-4} M) under 365 nm UV lamp of the transition process.

5. FT-IR Data

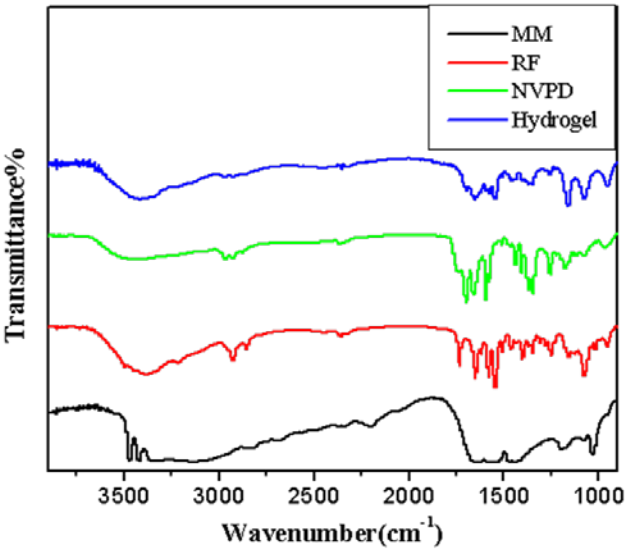

Figure S5. FT-IR spectra diluted with KBr for a NVPD/RF /MM= 1/2/2 xerogel (blue), NVPD (green), MM (black), RF (red).

6. Optical Images of the Different Mole Ratio Gel Reversible Process by Adjusting the pH

a b c

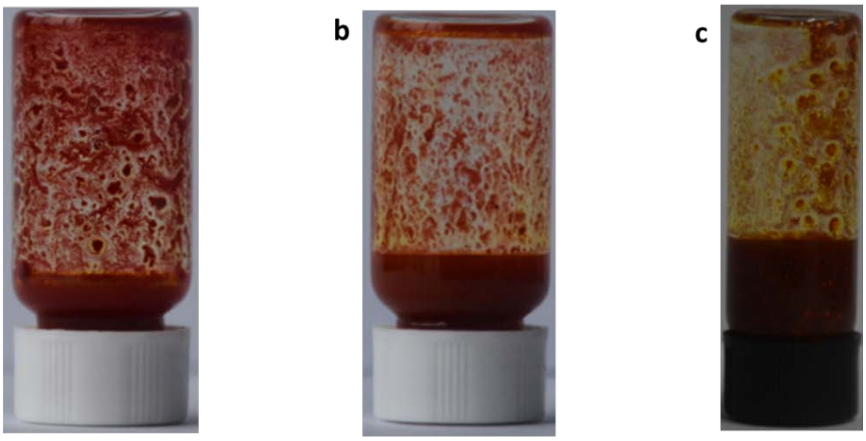

Figure S5. Optical images of the multicomponent gel for different ratio reversible process. (**a**) NVPD:RF:MM = 1:0.3:2; (**b**) NVPD:RF:MM = 1:1:2; (**c**) NVPD: RF:MM = 1:2:3.
